# Comparison of the Solution and Vacuum-Processed Squaraine:Fullerene Small-Molecule Bulk Heterojunction Solar Cells

**DOI:** 10.3389/fchem.2018.00412

**Published:** 2018-09-11

**Authors:** Guo Chen, Zhitian Ling, Bin Wei, Jianhua Zhang, Ziruo Hong, Hisahiro Sasabe, Junji Kido

**Affiliations:** ^1^Key Laboratory of Advanced Display and System Applications, Ministry of Education, Shanghai University, Shanghai, China; ^2^Department of Organic Device Engineering, Graduate School of Engineering, Research Center for Organic Electronics, Yamagata University, Yonezawa, Japan

**Keywords:** organic solar cells, bulk heterojunction, squaraine dye, solution-process, vacuum-process

## Abstract

Squaraine dyes have shown promising properties for high performance organic solar cells owing to their advantages of intense absorption and high absorption coefficients in the visible and near-infrared (NIR) regions. In this work, to directly compare the photovoltaic performance of solution- and vacuum-processed small-molecule bulk heterojunction (SMBHJ) solar cells, we employed a squaraine small molecular dye, 2,4-bis[4-(N,N-diisobutylamino)-2,6-dihydroxyphenyl] squaraine (DIBSQ), as an electron donor combined with fullerene acceptors to fabricate SMBHJ cells either from solution or vacuum deposition process. The solution-processed SMBHJ cell possesses a power conversion efficiency (PCE) of ~4.3%, while the vacuum-processed cell provides a PCE of ~6.3%. Comparison of the device performance shows that the vacuum-processed SMBHJ cells possess higher short-circuit current density, fill factor and thus higher PCE than the solution-processed devices, which should be assigned to more efficient charge transport and charge extraction in the vacuum-processed SMBHJ cells. However, solution-processed SMBHJ cells demonstrate more pronounced temperature-dependent device performance and higher device stability. This study indicates the great potential of DIBSQ in photovoltaic application via both of solution and vacuum processing techniques.

## Introduction

Organic solar cells (OSCs) have attracted much attention as a green solar energy technology for cost-effective, renewable energy sources because of their advantages of low-cost, light-weight, large-area manufacturing, and mechanical flexibility (Yu et al., 1995; Günes et al., 2007; Li et al., 2012; He et al., 2015; Duan et al., 2017). An efficient OSC must absorb over a broad spectral range from visible to near-infrared (NIR) wavelengths (350~950 nm) and convert the incident light effectively into charges (Yu et al., 1995). Bulk heterojunction (BHJ) cell with broad spectral absorbing photoactive layer has great potential to realize high power conversion efficiency (PCE) because the donor/acceptor system in this kind of device can efficiently overcome the strong exciton binding energy and thus achieve efficient charge generation (Lin et al., 2012; Chen et al., 2017b; Tang et al., 2017; Duan et al., 2018; Qi et al., 2018). Generally, there are two processing techniques to fabricate BHJ cells, i.e., solution processing and vacuum evaporation. These two deposition routes require the raw materials with much different properties, in particular solubility and thermal stability, respectively (Kronenberg et al., 2010; Hu et al., 2017). In general, the conjugated polymers with high solubility are deposited by solution process while small molecules (SMs) with high-thermal stability are deposited by vacuum evaporation. Solution processing is a relatively low-cost and fast method for depositing thin films. However, the stacking of multilayer layers becomes a great challenge because the interface erosion issue between the different solution-processed stacked layers (Hu et al., 2017). In comparison, vacuum deposition has several exceptive advantages such as accurate control of the thin film thickness and evaporation rate of the materials, and easy fabrication of multilayer devices by successive deposition of the materials. However, the vacuum conditions lead to high production costs (Kronenberg et al., 2010).

Recently, much effort has been dedicated to develop NIR absorbing polymer or small molecular donors combined with the fullerene acceptors to construct broad spectral absorbing BHJ layers (Li, 2012; Roncali et al., 2014; Chen et al., 2016b; Huang et al., 2016; Jiang et al., 2016; Du et al., 2018). Squaraine (SQ) dye is a kind of NIR absorbing small molecular donor material due to its advantages of simple synthetic route, high photochemical and thermal stability and high absorption coefficient in the NIR region (Ajayaghosh, 2005; Sasabe et al., 2014; Chen et al., 2015, 2017b). More recently, some NIR absorbing SQ donor materials with symmetric or asymmetrical molecular structures have been developed for high-performance BHJ cells by several groups: Silvestri et al. firstly introduced the hydrazine end-capped symmetric SQ donor into the BHJ system combined with [6,6]-phenyl-C61-butyric acid methyl ester (PCBM) acceptor and obtained a PCE of over 1% (Silvestri et al., 2008), and 2% of PCE was then obtained from the alkenyl-functionalized symmetric SQ and [6,6]-phenyl-C71-butyric acid methyl ester (PC_71_BM) based BHJ cell (Bagnis et al., 2010). Würthner's group reported several symmetric SQs based BHJ cells and achieved a PCE of 1.8% with unusually high short circuit current (*J*_sc_) of 12.6 mA/cm^2^ (Mayerhöffer et al., 2009). Wei et al. reported PCEs of over 5% from symmetric SQ and PC_71_BM based BHJ cells (Wei et al., 2011). Chen et al. further promoted the PCE of symmetric SQ based BHJ up to over 6% by using co-evaporation technology (Chen et al., 2012a). Huang's group has synthesized a series of NIR absorbing asymmetric SQ donors combined with PC_71_BM acceptor to realize PCEs of over 6% (Wu et al., 2018).

Among all the above mentioned SQ dyes, the symmetric SQ dye 2,4-bis[4-(N,N-diisobutylamino)-2,6-dihydroxyphenyl] squaraine (DIBSQ) has shown promising properties for highly efficient BHJ cells (Chen et al., 2012a): DIBSQ has a wide absorption spectra in the NIR region with high absorption coefficient of over 3 × 10^5^ cm^−1^, and its highest occupied molecular orbital (HOMO) and lowest unoccupied molecular orbital (LUMO) levels are located at −5.3 and −3.5 eV, respectively (Chen et al., 2016a), which provides a higher photocurrent and higher *V*_oc_ for the BHJ cells. The most unique property of DIBSQ is that the DIBSQ thin film can be deposited by using both of vacuum evaporation and solution processing technologies because the DIBSQ has high thermal stability and moderate solubility in organic solvents. To the best of our knowledge, it is rare that a small molecular thin film can be fabricated by using both of solution process and vacuum evaporation techniques (Chen et al., 2013). This unique property of DIBSQ indicates the photoactive layers of the DIBSQ:Fullerene based SMBHJ cells can be fabricated by spin-coating DIBSQ:PC_71_BM mixed solution or by vacuum co-evaporation of DIBSQ and C_70_, which offers the possibility to directly compare the device performance of two processing techniques.

In this work, to directly compare the two processing techniques for BHJs, i.e., solution process and vacuum evaporation process, we fabricated a DIBSQ:PC_71_BM SMBHJ cell by using spin-coating processing and a DIBSQ:C_70_ SMBHJ cell by using vacuum co-evaporation processing. Then the device performance and film properties of the SMBHJ active layers were characterized and systematically compared.

## Experimental section

### Materials

DIBSQ was synthesized according to the procedure reported by Tian et al. (2002), The purity of DIBSQ was proved to be over 99.9% by NMR and elemental analysis. MoO_3_, bathocuproine (BCP) and PC_71_BM were commercially available and used as received. Commercially available C_70_ was sublimated 3 times before use. Patterned indium-tine-oxide (ITO) glass substrates were successively cleaned by using detergent, deionized water, acetone and isopropanol in ultrasonic bath, respectively, finally the cleaned ITO substrates were kept in an oven at 80°C for 12 h to be completely dried.

### Film characterization

UV-vis absorption spectra were carried out using a UV-vis-NIR spectrophotometer (SHIMADZU, UV-3150). Atomic force microscopy (AFM) images were performed in air on a scanning probe microscope (Nanonavi SPA-400SPM, Japan) using a tapping mode. X-ray diffraction (XRD) patterns were measured using a high-resolution XRD diffractometer (SmartLab, Rigaku Co.). The films for these measurements were prepared by using the same fabrication conditions for the devices to enable accurate comparisons.

### Device fabrication and characterization

**Figure 1:**
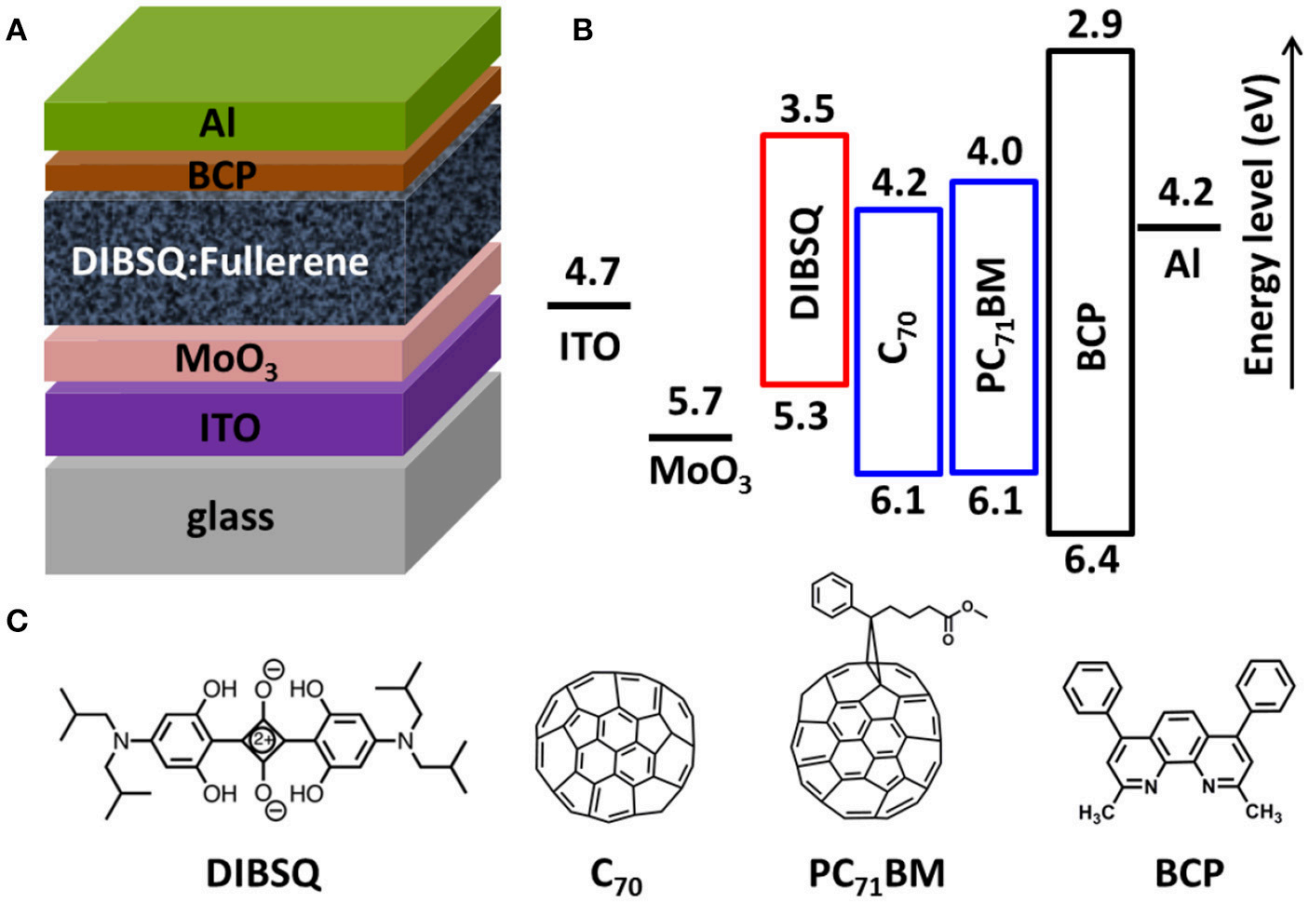
**(A)**Device architecture of the DIBSQ:Fullerene bulk heterojunction photovoltaic cells, i.e., ITO/MoO_3_ (5 nm)/DIBSQ:Fullerene (1:5, 60 nm)/BCP (10 nm)/Al (100 nm); **(B)** energy-level diagram, and **(C)** molecular structures of the materials under investigation.

The DIBSQ:PC_71_BM BHJ cells with the structure of ITO/MoO_3_ (5 nm)/DIBSQ:PC_71_BM (60 nm, 1:5)/BCP (10 nm)/Al (100 nm) (as shown in Figure 1) were fabricated as follows: the cleaned ITO substrates were exposed to UV ozone for 30 min and immediately transferred into a high-vacuum chamber for deposition of 5 nm-thick MoO_3_ at a base pressure of 1 × 10^−5^ Pa. The substrates were then transferred into a nitrogen-filled glove box, 60 nm-thick photoactive layers were fabricated by spin-coating DIBSQ:PC_71_BM solution (20 mg mL^−1^ in chloroform with donor:acceptor weight ratio of 1:5) on the MoO_3_ coated ITO surface at a rate of 4,000 revolutions per minute (rpm), then the DIBSQ:PC_71_BM blend films were thermally annealed at 70°C for 10 min. Finally, the substrates were transferred back to the high-vacuum chamber where 10 nm-thick BCP and 100 nm-thick Al cathode were successively deposited. The DIBSQ:C_70_ BHJ cells with the structure of ITO/MoO_3_ (5 nm)/DIBSQ:C_70_ (60 nm, 1:5)/BCP (10 nm)/Al (100 nm) were fabricated in a high vacuum chamber with a pressure of 5 × 10^−6^ Pa, where 5 nm MoO_3_ was deposited on the pre-cleaned ITO surface, then the 60 nm-thick DIBSQ:C_70_ photoactive layer were prepared by co-depositing DIBSQ and C_70_ with a blending ratio of 1:5, finally the devices were completed by evaporating 10 nm-thick BCP and 100 nm-thick Al cathode. The active area of solar cells is 0.04 cm^2^. Hole-only devices with the structure of ITO/MoO_3_ (5 nm)/DIBSQ:PC_71_BM (60 nm, 1:5) or DIBSQ:C_70_ (60 nm, 1:5)/MoO_3_ (5 nm)/Al (100 nm) and electron-only devices with the structure of ITO/Cs_2_CO_3_ (1 nm)/DIBSQ:PC_71_BM (60 nm, 1:5) or DIBSQ:C_70_ (60 nm, 1:5)/BCP (10 nm)/Al (100 nm) were fabricated to characterize hole and electron mobility in photoactive blend films by using space-charge-limited current (SCLC) method (Shrotriya et al., 2006), respectively. The current density-voltage (*J*-*V*) and external quantum efficiency (EQE) of BHJ cells were measured on a CEP-2000 integrated system (Bunkou Keiki Co.) under standard measurement conditions. The device performance data were averaged from 16 individually fabricated BHJ cells.

## Results and discussion

Figure 1 represents the architectures of the DIBSQ:Fullerene SMBHJ cells (Figure 1a), the corresponding energy-level diagram (Figure 1b) and the molecular structures of the organic materials used in the device (Figure 1c). In the SMBHJ device, MoO_3_ is adopted as hole-transporting layer. As demonstrated in our previous research (Chen et al., 2012b), the MoO_3_ layer effectively deepened the work function of ITO to −5.7 eV, and thus delivered a larger open circuit voltage (*V*_oc_) for the DIBSQ:Fullerene SMBHJ cells compared with those based on the typical ITO/PEDOT:PSS anode. The DIBSQ:Fullerene blend film is employed as the photoactive layer, while the bathocuproine (BCP) is used as electron-transporting layer. For comparison, the same ITO/MoO_3_ (5 nm) cathode and BCP (10 nm)/Al (100 nm) anode were employed in all devices, as well as the film thickness and the blend ratio of DIBSQ and Fullerene for all the devices were also kept same as 60 nm and 1:5 ratio, respectively. The only difference is the fabrication processing and the corresponding acceptor materials used for the photoactive layers: the solution-processed DIBSQ:PC_71_BM SMBHJ cells were prepared by spin-coating the DIBSQ:PC_71_BM blend solution, while the vacuum-processed DIBSQ:C_70_ SMBHJ cells were fabricated by co-evaporating the DIBSQ and C_70_ in the vacuum condition.

**Figure 2:**
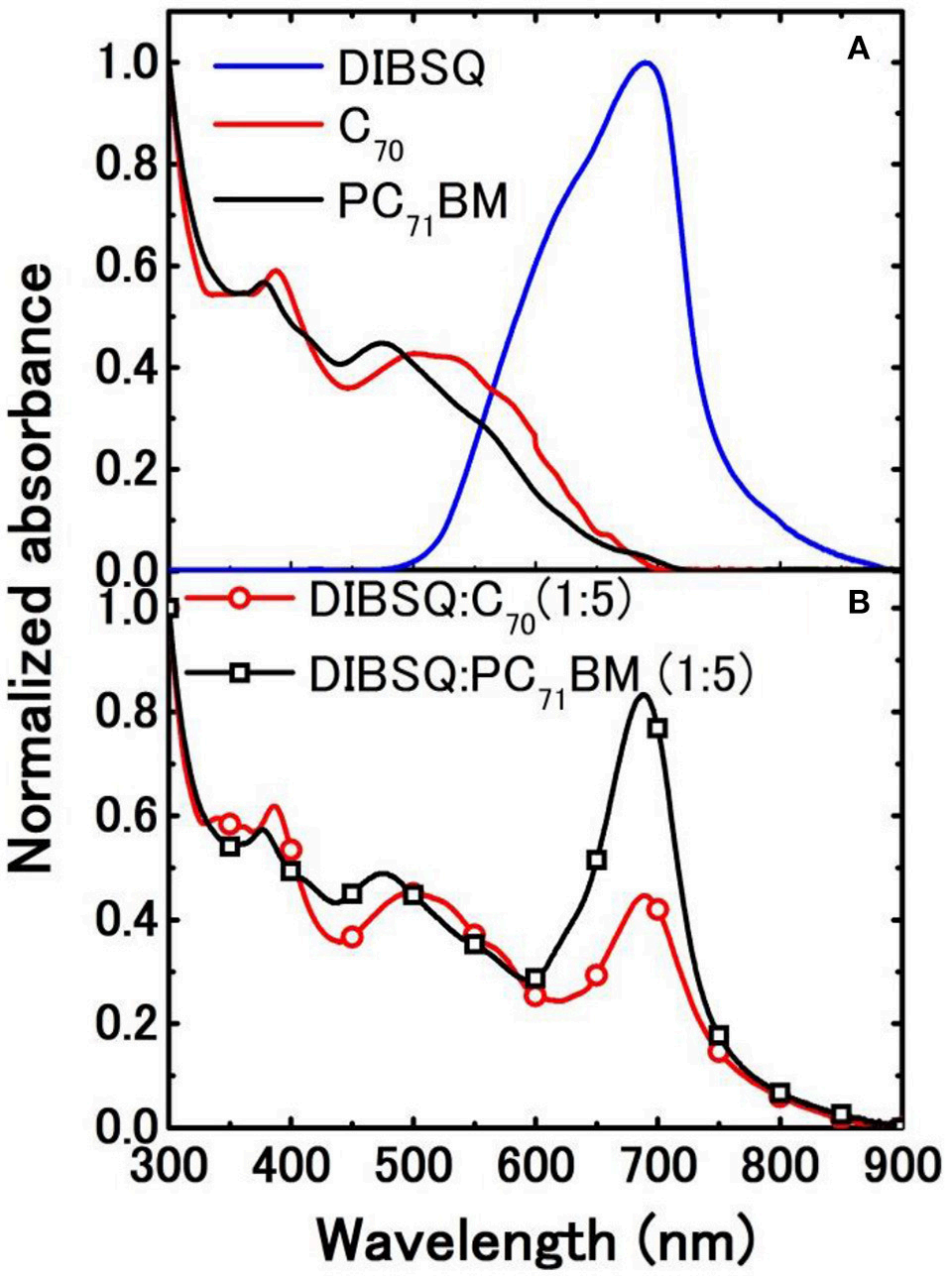
Absorbance of **(A)** DIBSQ, C_70_ and PC_71_BM neat films and **(B)** DIBSQ:PC_71_BM and DIBSQ:C_70_ blend films with blend ratio of 1:5.

The UV-Vis absorption spectra of the neat films of DIBSQ, C_70_, PC_71_BM and the blend films of DIBSQ:PC_71_BM and DIBSQ:C_70_ were characterized. As shown in Figure 2a, the DIBSQ film displays an absorption band in the region of 500–800 nm with an absorption peak at 700 nm; the C_70_ and PC_71_BM film show wide absorption bands between 300 and 740 nm, thus the blend films of both DIBSQ:PC_71_BM and DIBSQ:C_70_ display strong and broad absorption covering the region from visible to NIR, which leads to larger light-harvesting, and thus potentially obtains higher *J*_sc_ of the DIBSQ:Fullerene SMBHJ cells (Chen et al., 2014).

**Figure 3:**
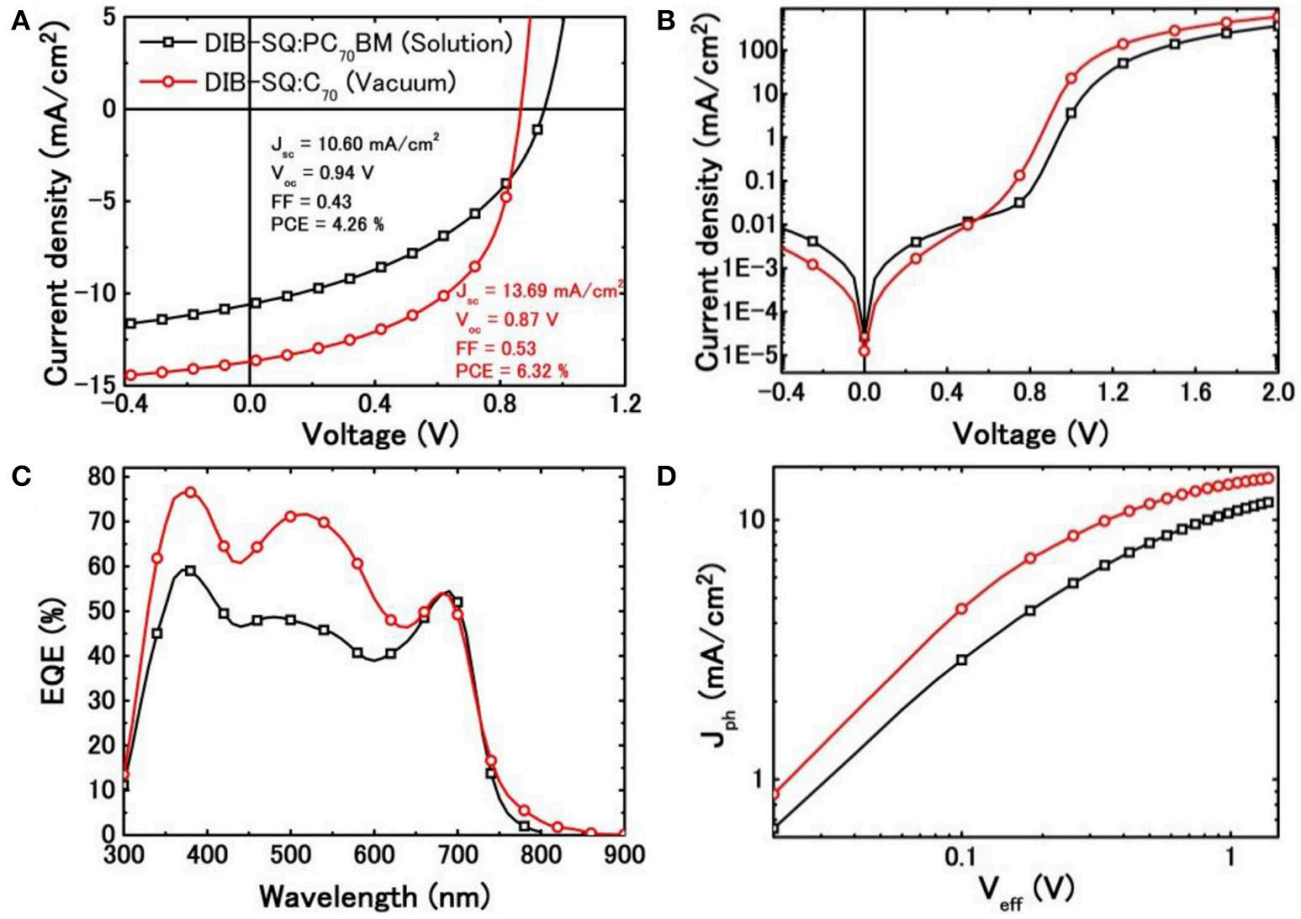
**(A)** *J*-*V* curves under AM 1.5G solar spectrum at 100 mW/cm^2^ illumination; **(B)** *J*-*V* curves under dark condition; **(C)** EQE spectra, and **(D)** *J*_ph_-*V*_eff_ curves measured at the AM1.5G condition (100 mW/cm^2^) of solution-processed DIBSQ:PC_71_BM and vacuum-processed DIBSQ:C_70_ SMBHJ cells.

Figure 3a exhibits the *J*-*V* curves of the SMBHJ cells under 100 mW/cm^2^ AM 1.5 illumination. As shown in Figure 3a and Table 1, the solution-processed DIBSQ:PC_71_BM SMBHJ cell possesses a PCE of 4.26% with a *J*_sc_ of 10.60 mA/cm^2^, a *V*_oc_ of 0.94 V and a fill factor (FF) of 0.43. While the vacuum-processed DIBSQ:C_70_ SMBHJ cell provides a PCE of 6.32% with a *J*_sc_ of 13.69 mA/cm^2^, *V*_oc_ of 0.87 V and FF of 0.53. Comparison of the device performance shows that the vacuum-processed BHJ cells possess higher *J*_sc_ and FF and thus higher PCE than the solution-processed devices, which should be assigned to more efficient charge transport and charge extraction in the vacuum-processed SMBHJ cells owing to much higher and more balanced hole and electron mobilities in their active layers (Table 1) (Chen et al., 2017b), as discussed in a subsequent section. The lower *V*_oc_ in the vacuum-processed DIBSQ:C_70_ SMBHJ cells can be explained by the deeper LUMO level of C_70_ acceptor compared with that of PC_71_BM acceptor, as shown in Figure 1b. Normally, the *V*_oc_ of BHJs depends on the energy level difference between the HOMO of the donor (HOMO _Donor_) and LUMO of the acceptor (LUMO _Acceptor_) (Chen et al., 2017b), the deeper LUMO level of C_70_ leads to smaller |HOMO _Donor_| – |LUMO _Acceptor_| value and thus smaller *V*_oc_. As shown in Table 1, comparing with the solution-processed DIBSQ:PC_71_BM SMBHJ cell, the series resistance (*R*_s_) of the DBSQ:C_70_ SMBHJ cell obviously decreases from 16.2 to 6.8 Ω cm^2^, whereas the shunt resistance (*R*_sh_) increase from 2.9 × 10^3^ to 4.0 × 10^3^ Ω cm^2^ in the same time. The decreased *R*_s_ and increased *R*_sh_ also contribute to higher FF of the vacuum-processed DIBSQ:C_70_ SMBHJ device (Chen et al., 2017b).

**Table 1:** Comparison of device performance of solution-processed DIBSQ:PC_71_BM and vacuum-processed DIBSQ:C_70_ SMBHJ cells.

**Fabrication processing**	***J*_sc_ (mA/cm^2^)**	***V*_oc_ (V)**	**FF**	**PCE (%)**	**μ_h_ (cm^2^/Vs)^a^**	**μ_e_ (cm^2^/Vs)^a^**	***R*_s_ (Ω cm^2^)**	***R*_sh_ (Ω cm^2^)**
Solution	10.60	0.94	0.43	4.26	2.1 × 10^−5^	3.7 × 10^−4^	16.2	2.9 × 10^3^
Vacuum	13.69	0.87	0.53	6.32	9.8 × 10^−5^	6.8 × 10^−4^	6.8	4.0 × 10^3^

a *Carrier mobility data were originated from a space-charge-limited current (SCLC) model*.

*The device performance data were averaged from 16 individually fabricated BHJ cells*.

Figure 3b displays the *J*-*V* curves of the SMBHJ cells under dark condition. The solution-processed DIBSQ:PC_71_BM SMBHJ cell demonstrates a turn-on voltage of approximately 0.8–1.0 V while it is approximately 0.7–0.9 V for the vacuum-processed DIBSQ:C_70_ device, which indicates that the superior limit of the attainable *V*_oc_ in SMBHJ cell, i.e., the built-in potential (*V*_bi_) across the SMBHJ cell, obviously decreases by using the vacuum-processed DIBSQ:C_70_ active layer (He et al., 2011; Chen et al., 2017b). This observation means that solution-processed DIBSQ:PC_71_BM SMBHJ device possesses larger *V*_bi_ than the vacuum-processed DIBSQ:C_70_ SMBHJ device, which should be ascribed to poorer charge-transporting properties in the solution-processed SMBHJ cells. As shown in Figure 3c, the EQE spectra of both devices consist of three major peaks at 370, 500, and 700 nm approximately. The first two peaks located at 370 and 500 nm correspond to the absorption of PC_71_BM or C_70_, and the third peak at 700 nm is from the absorption of DIBSQ, which is consistent with the absorption spectra of the blend DIBSQ:PC_71_BM and DIBSQ:C_70_ films (Figure 2b). Even though the photo response in the longer wavelength region are almost same for the solution- and vacuum-processed SMBHJ cells, the photo response in the near ultraviolet and visible region for the vacuum-processed DIBSQ:C_70_ SMBHJ cell is significantly enhanced up to 76 and 72%, respectively, which explains that why the vacuum-processed SMBHJ cell possesses higher *J*_sc_ than that of solution-processed SMBHJ cell. The normalized EQE spectra (Figure S1) shows that the photo response in the longer wavelength region of the solution-processed DIBSQ:PC_71_BM SMBHJ cell is much stronger than that of the DIBSQ:C_70_ SMBHJ cell, which is consistent with the observation in the normalized absorption spectra of DIBSQ:Fullerene films (Figure 2b). Moreover, the obviously higher and longer photo response from 750 to 850 nm should be ascribed to the absorption of charge transfer states between DIBSQ and C_70_ (Wang et al., 2013), which also contributes to the higher *J*_sc_ in the DIBSQ:C_70_ SMBHJ cell.

To further study the effect of fabrication processing on the SMBHJ device performance, we also characterized the photocurrent density (*J*_ph_) vs. effective voltage (*V*_eff_) of the DIBSQ:PC_71_BM and DIBSQ:C_70_ SMBHJ cells, respectively, as demonstrated in Figure 3d. Here, *J*_ph_ and *V*_eff_ are defined by

(1) $$\begin{array}{r} J_{\text{ph}}=J_{\text{L}}-J_{\text{D}} \end{array}$$

(2) $$\begin{array}{r} V_{\text{eff}}=V_{0}-V_{\text{a}} \end{array}$$

where J_L_ and J_D_ represent the current density measured under AM 1.5G illumination and in the dark at an applied bias voltage *V*_b_, respectively. *V*_0_ is the built-in potential, which can by identified as the voltage at *J*_ph_ = 0 (Wu et al., 2016). Figure 3d displays that the *J*_ph_ increases linearly with *V*_eff_ at low *V*_eff_ range (< 0.1 V), and then gradually saturates at a high *V*_eff_. In general, it is supposed that all photogenerated excitons are dissociated into free carriers and extracted by electrodes at high *V*_eff_ (Chen et al., 2017b). At lower *V*_eff_ region, higher *J*_ph_ can be found for the solution-processed SMBHJ device than that of the vacuum-processed device, which reflects the charge collection in the vacuum-processed SMBHJ cells is more efficient than that in the solution-processed SMBHJ cells. The enhanced charge collection efficiency leads to improved device performance in the vacuum-processed devices, as discussed in the previous sections.

**Figure 4:**
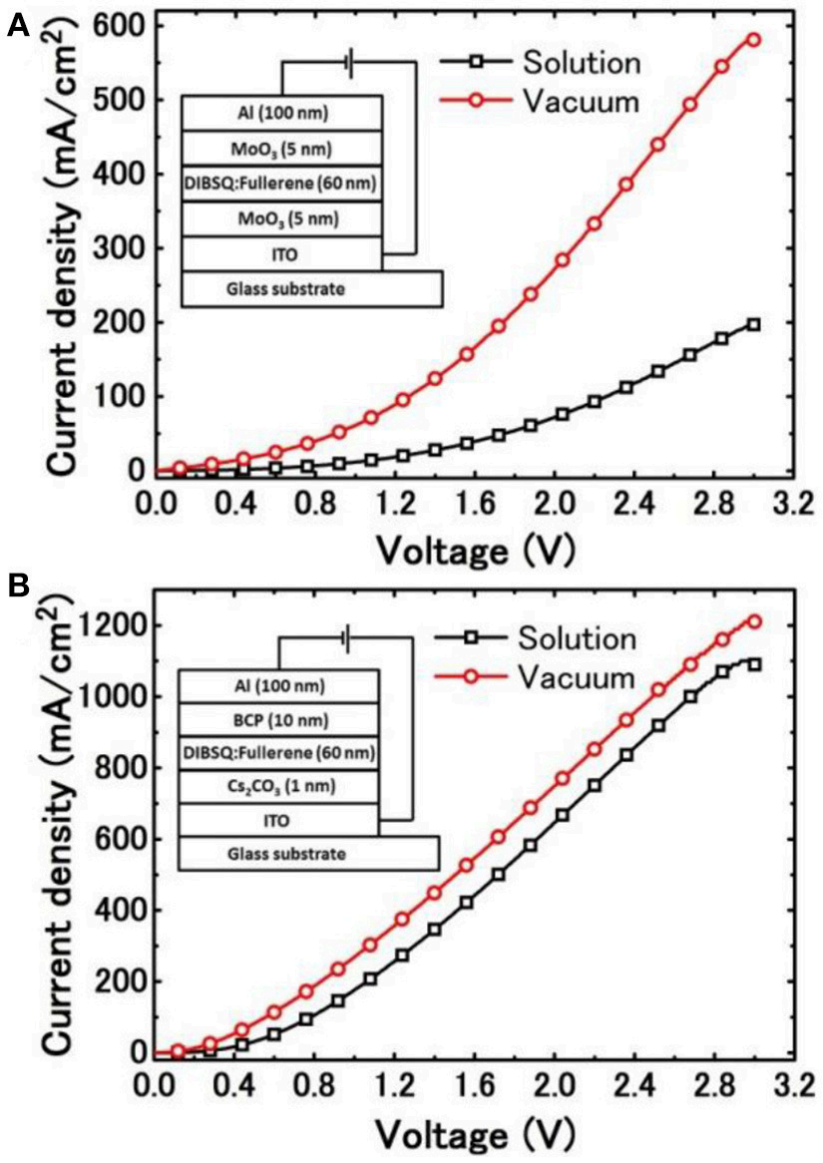
*J*-*V* characteristics of **(A)** hole-only devices and **(B)** electron-only devices based on solution-processed DIBSQ:PC_71_BM and vacuum-processed DIBSQ:C_70_ blend films, inset show the schematic structures of the corresponding single carrier devices.

Carrier mobilities in BSQ:Fullerene blend films deposited from different fabrication processing were also characterized and compared, as shown in Figure 4. Hole-only diodes were fabricated using the structure of ITO/MoO_3_ (5 nm)/DIBSQ:PC_71_BM (60 nm, 1:5) or DIBSQ:C_70_ (60 nm, 1:5)/MoO_3_ (5 nm)/Al (100 nm), while the electron-only diodes were prepared using the structure of ITO/Cs_2_CO_3_ (1 nm)/DIBSQ:PC_71_BM (60 nm, 1:5) or DIBSQ:C_70_ (60 nm, 1:5)/BCP (10 nm)/Al (100 nm) for electrons. Where the DIBSQ:PC_71_BM and DIBSQ:C_70_ blend films were prepared by using the same solution- and vacuum-processing, respectively, as those in the SMBHJ devices. The *J*-*V* curves of the hole- and electron-only diodes were characterized as shown in Figure 4, and then the carrier mobilities were calculated by fitting the *J*-*V* data using the SCLC method according to the following equation (3) (Wang et al., 2015):

(3) $$\begin{array}{r} J=\frac{9}{8}\mu \varepsilon_{0}\varepsilon_{r}\frac{V^{2}}{d^{3}} \end{array}$$

where *J* represents the current density, μ represents effective carrier mobility, ε_0_ and ε_r_ represent the absolute dielectric constant and relative dielectric constant, respectively, *d* represents the thickness of the DBSQ:PC_71_BM film and *V* is the applied voltage (Chen et al., 2017b). The SCLC-estimated hole and electron mobility data were summarized in Table 1. The hole and electron mobility in vacuum-processed DIBSQ:C_70_ films are about 4.7 and 1.8 times greater than that in the solution-processed DBISQ:PC_71_BM film, respectively, which shall be ascribed to the closer intermolecular distance in the denser vacuum-processed DIBSQ:C_70_ film. Meanwhile the hole and electron mobilities in vacuum-processed DIBSQ:C_70_ films are much more balanced than those in solution-processed DBISQ:PC_71_BM films. More balanced hole and electron mobilities leads to more efficient charge extraction and thus much higher device performance in the DIBSQ:C_70_ SMBHJ devices (Chen et al., 2017b).

**Figure 5:**
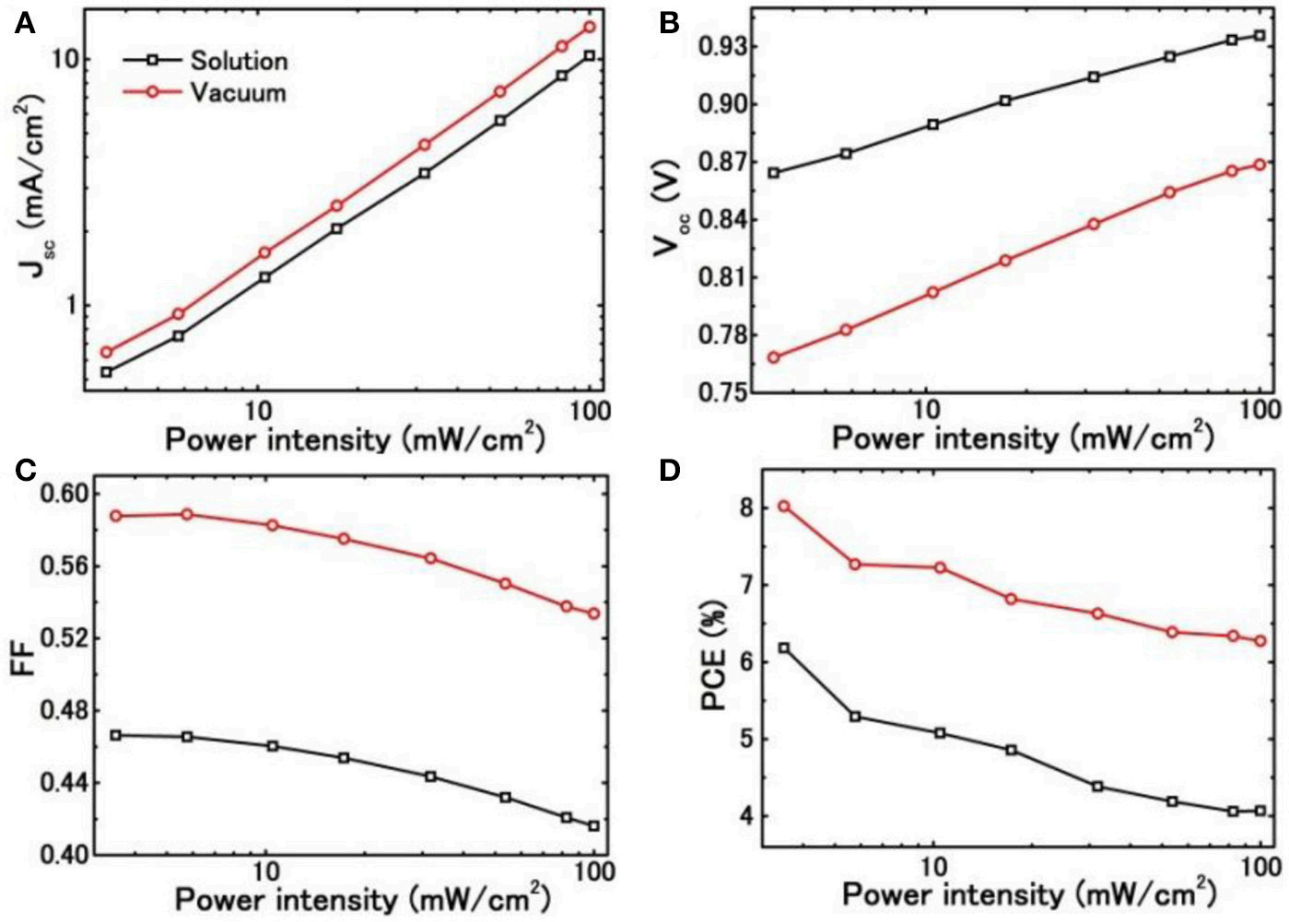
**(A)** The short circuit current (*J*_sc_); **(B)** open circuit voltage (*V*_oc_); **(C)** fill factor (FF) and **(D)** power conversion efficiency (PCE) vs. power intensity for solution-processed DIBSQ:PC_71_BM SMBHJ cells and vacuum-processed DIBSQ:C_70_ SMBHJ cells.

The light-intensity dependence of device performance for the DIBSQ:Fullerene SMBHJ cell was also characterized and compared, as shown in Figure 5. For both of the solution- and vacuum SMBHJ cells, the *J*_sc_ is linearly proportional to the light intensity. The slope value (0.91) nears the ideal value (1.0) for OSC device. The *V*_oc_ of the device shows a sub-linear trend with saturation when the light intensity reaches 100 mW/cm^2^. The slope is roughly close to *kT/q*, demonstrating that bimolecular recombination dominates, where *k* is Boltzmann's constant, *T* is the temperature, and *q* is elementary charge (Koster et al., 2005; Chen et al., 2017a). The FF decreases with increasing the light intensity, which indicates that the recombination loss of the device is sensitive to both carrier density and electrical field. The PCEs of the solution-processed DIBSQ:PC_71_BM cell and DIBSQ:C_70_ SMBHJ cell improve up to 6.21 and 8.06% at 3.5 mW cm^−2^, respectively, respective to suppressed non-germinated recombination (Chen et al., 2012a), which is a strong indication of the great potential of the DIBSQ:Fullerene SMBHJ cell for the commercial application in the lower light intensity ambient.

**Figure 6:**
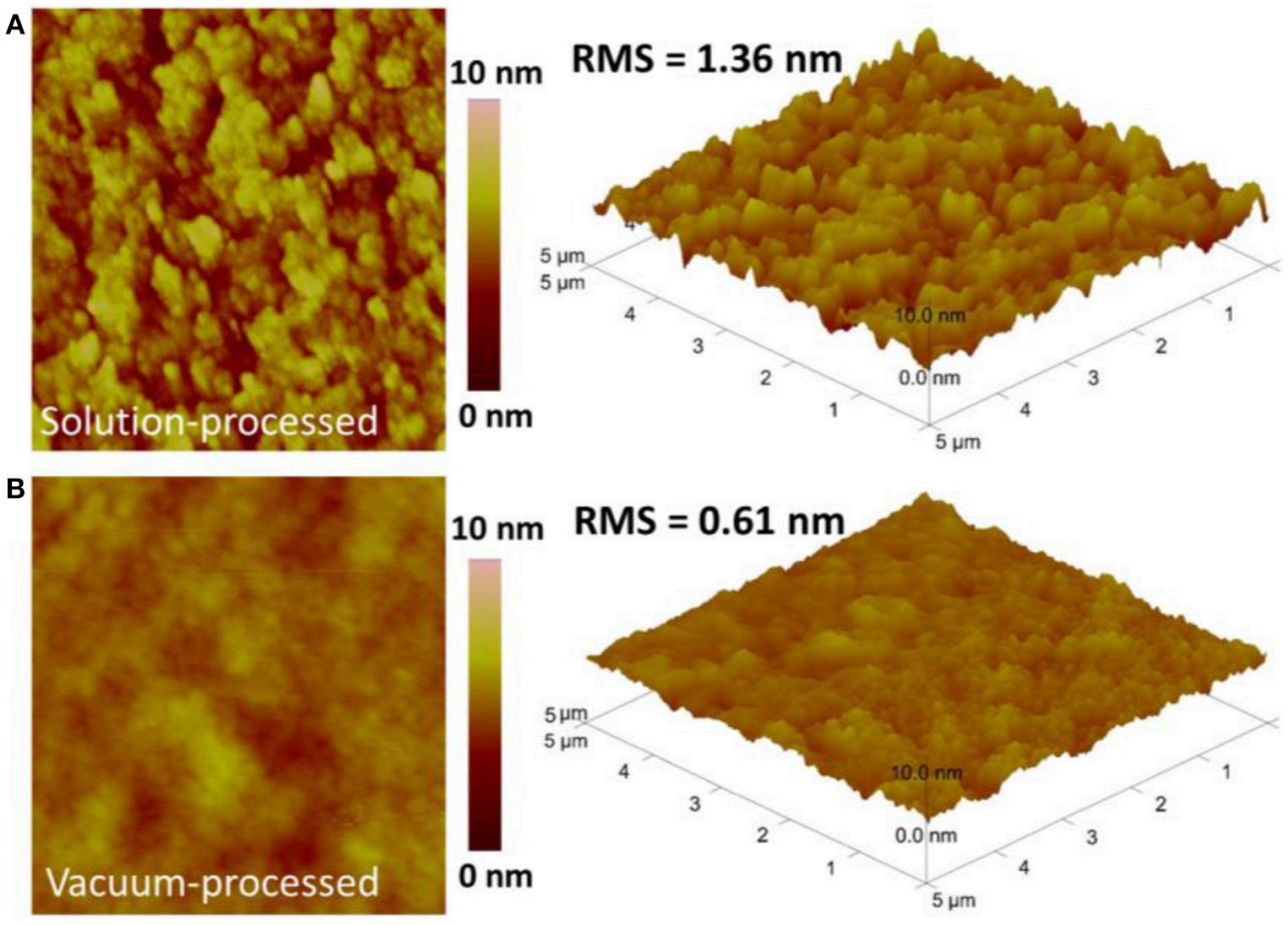
AFM topographic and 3D images of **(A)** the solution-processed DIBSQ:PC_71_BM (1:5, 60 nm) film and **(B)** the vacuum-processed DIBSQ:C_70_ (1:5, 60 nm) film.

To deeply understand the device performance of DIBSQ:Fullerene BHJ cells, we used AFM and XRD characterizations to study the film morphology of the BHJ active layer fabricated by using solution- or vacuum processing, as depicted in Figure 6, Figure S2. From the AFM images, a small root-mean-square (RMS) roughness of 1.36 and 0.61 nm for the solution-processed DIBSQ:PC_71_BM and vacuum-processed DIBSQ:C_70_ films were determined, respectively. And no peaks can be observed in the XRD patterns. These observations indicate that both of the solution- and vacuum-processed photoactive layers are amorphous. Though the amorphous feature of the photoactive layers is not conducive to charge transport, the small roughness of the photoactive layers are critical to avoid leakage current (Chen et al., 2012b), as demonstrated in Figure 3b.

**Figure 7:**
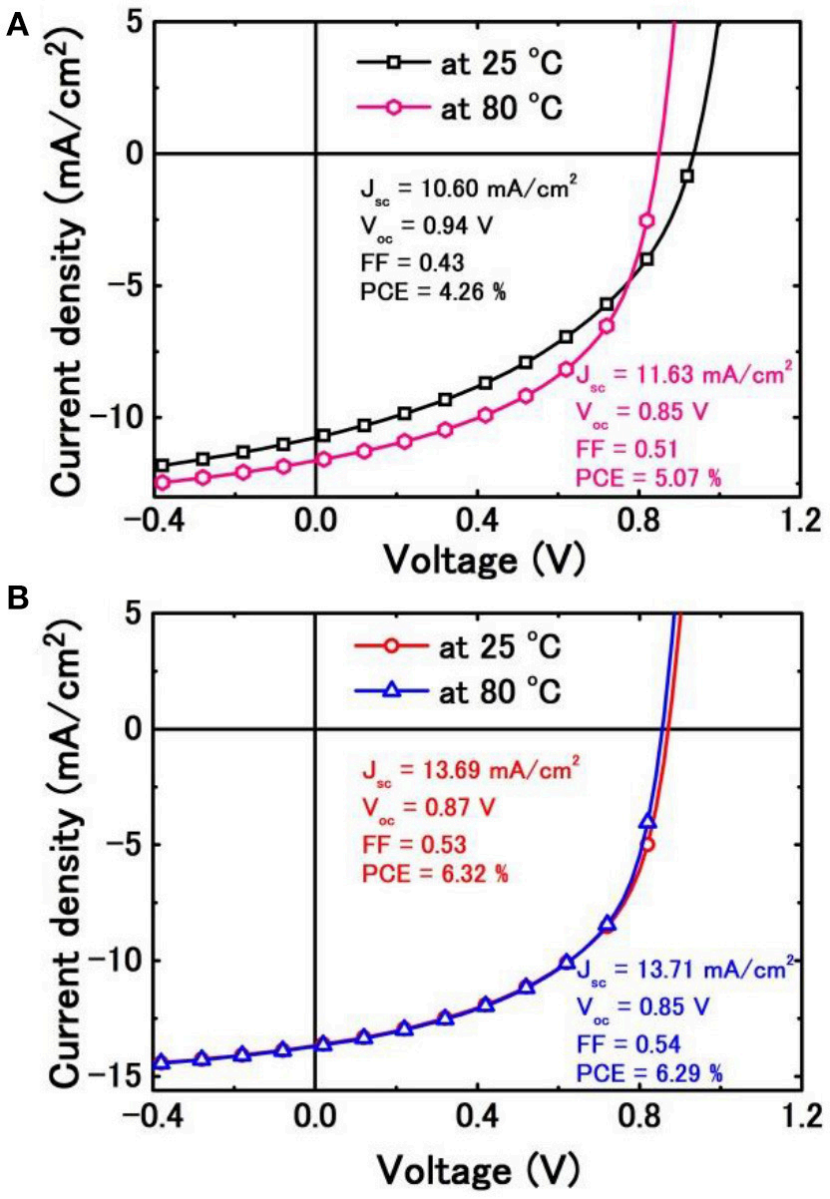
**(A)** *J*-*V* characteristics of the solution-processed DIBSQ:PC_71_BM SMBHJ cells at 25 and 80°C; and **(B)** *J*-*V* characteristics of the vacuum-processed DIBSQ:C_70_ SMBHJ cells at 25 and 80°C.

Considering DIBSQ:PC_71_BM SMBHJ cell has pronounced temperature-dependent performance, which has been systemically studied by our group (Chen et al., 2016a). In this work, we also test the device performance of vacuum-processed DIBSQ:C_70_ SMBHJ cell at higher temperature, and then compare the device performance with that of the solution-processed DIBSQ:PC_71_BM SMBHJ cell at the same testing temperature, as shown in Figure 7. For the solution-processed SMBHJ cell, the PCE will significantly increase up to 5.07% with increased *J*_sc_ and FF at a testing temperature of 80°C. While the *V*_oc_ decreases in the same time owing to the carrier recombination of the SMBHJ device (Chen et al., 2016a). As a result, the PCE is near 20% enhancement at 80°C comparing with the device performance at 25°C. However, we can not observe the similar temperature-dependent device performance from the vacuum-processed DIBSQ:C_70_ SMBHJ system. The DIBSQ:C_70_ SMBHJ cell at 80°C shows a slightly increased *J*_sc_ and FF while slightly decreased *V*_oc_ and thus a similar PCE compared with the device performance at 25°C, which can be explained by the almost unchanged carrier mobilities in the DIBSQ:C_70_ film compared with those at 25°C (Table S1). This observation is different from that in the DIBSQ:PC_71_BM SMBHJ cell, in which the carrier mobility will significant increase and thus the device performance will increase in the same time with increasing the testing temperature.

**Figure 8:**
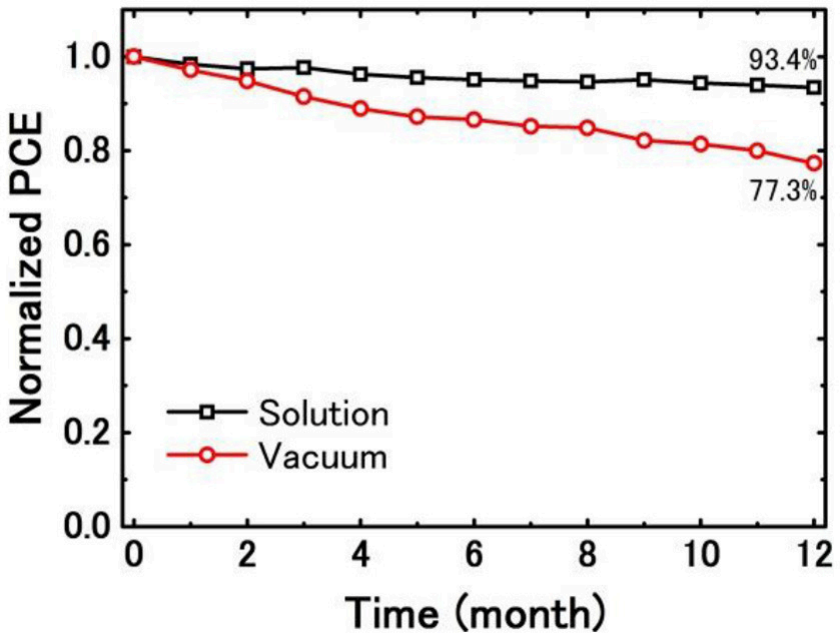
Comparison of the degradation of normalized PCE of solution-processed DIBSQ:PC_71_BM SMBHJ cells and vacuum-processed DIBSQ:C_70_ SMBHJ cells.

Besides PCE, stability is another important parameter to evaluate the performance of an OSC cell (Ecker et al., 2011). Figure 8 shows the degradation of normalized PCE of the solution- and vacuum-processed BHJ cells in 1 year, and all the measurements were carried out with all devices kept in air with glass encapsulation. After keeping in air one year, the PCE of the solution-processed DIBSQ:PC_71_BM SMBHJ cell still remains 93.4% of the initial value, while the PCE of the vacuum-processed DIBSQ:C_70_ SMBHJ cell remains 77.3% of the initial number. The higher device stability of the solution-processed DIBSQ:PC_71_BM SMBHJ cell demonstrates that the stable DIBSQ and PC_71_BM materials employed in the device can efficiently resist the chemical and photochemical degradation inside the SMBHJ cells, which is a very important property for the commercial application because many OSC materials can realize higher PCE while short lifetime.

## Conclusions

In summary, to directly compare the device performance of solution- and vacuum-processed SMBHJ solar cells, we employed a SQ dye, which can be deposited by using both of the solution and vacuum processing, as electron donor combined with fullerene as acceptor to construct solution- and vacuum-processed DIBSQ:Fullerene SMBHJ cells. Then the device performance were characterized and compared. The results demonstrates that the vacuum-processed cell provides a ~47% higher PCE than that of the solution-processed SMBHJ cell due to more efficient charge transport and charge extraction in the vacuum-processed SMBHJ cells. However, solution-processed SMBHJ cells demonstrate more pronounced temperature-dependent device performance and higher device stability. The light intensity-dependent device performance for both of the solution- and vacuum-processed DIBSQ:Fullerene SMBHJ cell indicates their promising application in the lower light intensity ambient. This study indicates the great potential of DIBSQ in photovoltaic application via both of solution and vacuum processing techniques.

## Author contributions

GC, HS, and JK designed experiments, GC and ZL carried out experiments, BW and ZH analyzed experimental results GC, JZ, and HS wrote the manuscript.

### Conflict of interest statement

The authors declare that the research was conducted in the absence of any commercial or financial relationships that could be construed as a potential conflict of interest.
