# Supplementary material for: Comparison of the Solution and Vacuum-Processed Squaraine:Fullerene Small-Molecule Bulk Heterojunction Solar Cells
Source: Front Chem. 2018 Sep 11;6:412. doi: 10.3389/fchem.2018.00412 (PMC6141623; doi:10.3389/fchem.2018.00412)
Supplement: Supplementary file 1 [file Image_1.pdf]

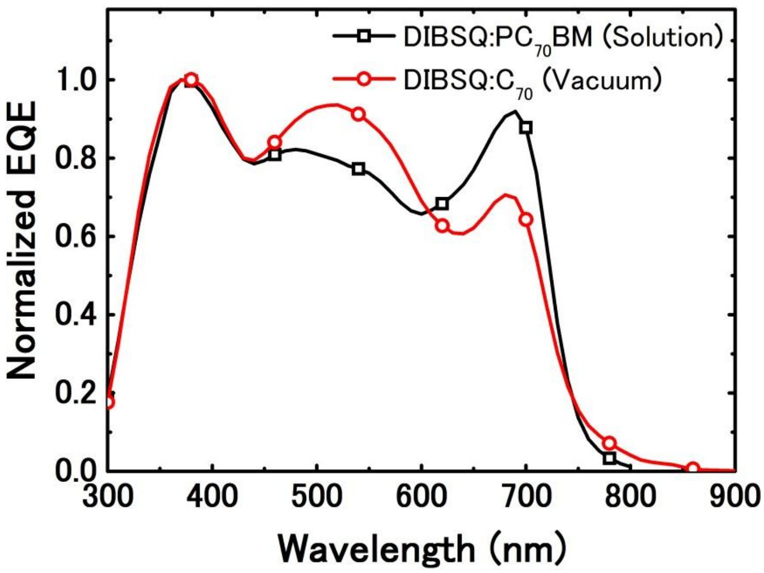

FIGURE S1 Normalized EQE spectra of the solution-processed DIBSQ:$PC_{71}BM$ and vacuum-processed DIBSQ:C_{70} SMBHJ cells
