# Supplementary material for: Comparison of the Solution and Vacuum-Processed Squaraine:Fullerene Small-Molecule Bulk Heterojunction Solar Cells
Source: Front Chem. 2018 Sep 11;6:412. doi: 10.3389/fchem.2018.00412 (PMC6141623; doi:10.3389/fchem.2018.00412)
Supplement: Supplementary file 3 [file Table_1.pdf]

Table S1. Carrier mobility data of vacuum-processed DIBSQ:C_{70} film derived from a space-charge-limited current (SCLC) method.

Temperature	μ_h (cm^2/Vs)	μ_e (cm^2/Vs)
25 °C	9.8×10^{-5}	6.8×10^{-4}
80 °C	1.0×10^{-4}	7.1×10^{-4}
